# Supplementary material for: Application of Ultrafiltration to Produce Sheep’s and Goat’s Whey-Based Synbiotic Kefir Products
Source: Membranes (Basel). 2023 Apr 28;13(5):473. doi: 10.3390/membranes13050473 (PMC10221410; doi:10.3390/membranes13050473)
Supplement: Supplementary file 1 [file membranes-13-00473-s001.zip › membranes-2370224-supplementary.pdf]

Table S1.-Color difference values (ΔEab^*) between different sheep's kefir products, and for the same product over storage.

	EK 1d	EKABT5 1d	TK 1d	TKABT5 1d	EK 10d	EKABT5 10d	TK 10d	TKABT5 10d	EK 20d	EKABT5 20d	TK 20d	TKABT5 20d	EK 30d	EKABT5 30d	TK 30d
**EKABT5 1d**	40.4														
**TK 1d**	18.3	4.6													
**TKABT5 1d**	0.8	31.3	12.7												
**EK 10d**	12.9	91.0	58.1	17.2											
**EKABT5 10d**	2.3	33.7	15.6	1.3	14.0										
**TK 10d**	6.5	43.8	24.7	5.4	10.5	1.7									
**TKABT5 10d**	8.2	74.0	45.1	10.9	1.0	7.9	5.2								
**EK 20d**	25.0	2.2	1.2	17.6	66.9	20.0	28.3	52.4							
**EKABT5 20d**	5.3	33.2	16.8	2.9	16.5	1.4	1.6	9.6	19.1						
**TK 20d**	24.9	13.5	10.8	17.1	50.9	14.3	15.4	37.9	7.7	10.2					
**TKABT5 20d**	12.5	15.7	7.5	7.4	34.8	5.5	7.6	24.3	7.6	4.1	2.2				
**EK 30d**	51.4	0.8	8.9	40.8	105.6	42.8	52.9	86.9	5.0	41.2	16.1	20.8			
**EKABT5 30d**	50.3	0.9	8.9	39.7	102.6	41.0	50.3	84.2	4.8	39.2	14.1	19.0	0.1		
**TK 30d**	59.1	2.3	12.9	47.4	114.2	48.5	57.9	94.6	7.8	46.2	17.3	23.7	0.5	0.4	
**TKABT5 30d**	60.2	2.2	12.9	48.5	117.2	50.4	60.6	97.4	7.9	48.1	19.2	25.5	0.4	0.5	0.1

Table S2.-Color difference values (ΔEab*) between different goat's kefir products, and for the same product over storage.

	EK 1d	EKABT5 1d	TK 1d	TKABT5 1d	EK 10d	EKABT5 10d	TK 10d	TKABT5 10d	EK 20d	EKABT5 20d	TK 20d	TKABT5 20d	EK 30d	EKABT5 30d	TK 30d
**EKABT5 1d**	2.6														
**TK 1d**	1.7	1.8													
**TKABT5 1d**	0.2	4.0	2.3												
**EK 10d**	2.3	8.8	4.2	1.5											
**EKABT5 10d**	2.5	9.2	5.0	1.7	0.1										
**TK 10d**	2.4	8.6	3.9	1.6	0.0	0.2									
**TKABT5 10d**	2.3	8.5	3.8	1.5	0.0	0.2	0.0								
**EK 20d**	3.7	11.4	6.0	2.5	0.2	0.2	0.2	0.2							
**EKABT5 20d**	2.2	8.5	4.2	1.4	0.0	0.1	0.0	0.0	0.2						
**TK2 0d**	3.4	11.1	6.0	2.4	0.2	0.1	0.3	0.3	0.0	0.2					
**TKABT5 20d**	3.7	11.6	6.4	2.6	0.2	0.2	0.3	0.3	0.0	0.2	0.0				
**EK 30d**	49.2	66.4	47.0	45.2	30.8	31.9	30.3	30.6	27.6	31.8	29.0	28.2			
**EKABT5 30d**	60.3	78.6	57.1	55.9	39.9	41.3	39.3	39.6	36.3	41.0	37.9	37.0	0.6		
**TK 30d**	59.9	78.9	57.5	55.3	39.3	40.6	38.8	39.1	35.6	40.4	37.1	36.2	0.6	0.1	
**TKABT5 30d**	57.6	76.3	55.4	53.1	37.4	38.7	36.9	37.3	33.8	38.5	35.4	34.4	0.4	0.2	0.0
